# High-Order Laplacian Regularized Low-Rank Representation for Multimodal Dementia Diagnosis

**DOI:** 10.3389/fnins.2021.634124

**Published:** 2021-03-12

**Authors:** Aimei Dong, Zhigang Li, Mingliang Wang, Dinggang Shen, Mingxia Liu

**Affiliations:** ^1^School of Computer Science and Technology, Qilu University of Technology (Shandong Academy of Science), Jinan, China; ^2^Department of Radiology and Biomedical Research Imaging Center, University of North Carolina at Chapel Hill, Chapel Hill, NC, United States; ^3^College of Computer Science and Technology, Nanjing University of Aeronautics & Astronautics, Nanjing, China; ^4^School of Biomedical Engineering, ShanghaiTech University, Shanghai, China; ^5^Shanghai United Imaging Intelligence Co., Ltd., Shanghai, China; ^6^Department of Artificial Intelligence, Korea University, Seoul, South Korea

**Keywords:** high-order, low-rank representation, dementia, classification, incomplete heterogeneous data

## Abstract

Multimodal heterogeneous data, such as structural magnetic resonance imaging (MRI), positron emission tomography (PET), and cerebrospinal fluid (CSF), are effective in improving the performance of automated dementia diagnosis by providing complementary information on degenerated brain disorders, such as Alzheimer's prodromal stage, i.e., mild cognitive impairment. Effectively integrating multimodal data has remained a challenging problem, especially when these heterogeneous data are incomplete due to poor data quality and patient dropout. Besides, multimodal data usually contain noise information caused by different scanners or imaging protocols. The existing methods usually fail to well handle these heterogeneous and noisy multimodal data for automated brain dementia diagnosis. To this end, we propose a high-order Laplacian regularized low-rank representation method for dementia diagnosis using block-wise missing multimodal data. The proposed method was evaluated on 805 subjects (with incomplete MRI, PET, and CSF data) from the real Alzheimer's Disease Neuroimaging Initiative (ADNI) cohort. Experimental results suggest the effectiveness of our method in three tasks of brain disease classification, compared with the state-of-the-art methods.

## 1. Introduction

Alzheimer's disease (AD) is a highly prevalent and severe irreversible neurodegenerative disease and it has already devastated millions of lives in the world (Cuingnet et al., 2011). AD is of an escalating epidemic and it is a tremendous challenge to global health care systems (Kuljis~, 2010). AD is the most common dementia among the elders and it accounts for about 60–80% among the age-related dementia cases. It is estimated that the regular cost for caring for AD patients from families and health-care systems is up to $100 million every year (Reiman et al., 2010). The number of AD patients increases very rapidly. It is estimated that the number of these patients nearly doubles every year and the number will be up to 115 million worldwide (Kuljis~, 2010) and 13.8 million in the United States (Association et al., 2013) by 2050. Clinic and research show that the potential pathology of AD appears many years ahead of the onset of cognitive symptoms. Therefore, extensive studies pay attention to the automated diagnosis of AD and progression prediction of its prodrome, i.e., mild cognitive impairment (MCI), to delay the progress of AD.

Three types of data modalities have been widely used to effectively predict the progression of AD, i.e., structural magnetic resonance imaging (MRI), fluorodeoxyglucose positron emission tomography (PET), and cerebrospinal fluid (CSF). Specifically, MRI provides anatomical information about the brain, and MRI-based feature representations (e.g., regional volumetric measures, cortical thickness, and connectivity information) can be used to quantify AD-associated brain abnormalities (Liu et al., 2015a, 2016). Also PET can be employed to detect the defect in cerebral metabolic rate for glucose in the human brain (Foster et al., 2007; Liu et al., 2015b). In addition, CSF is closely related to the cognitive decline in AD and MCI subjects (Hansson et al., 2006). It is of great value to capture the common hidden representation and complementary information among three modalities for AD diagnosis and MCI conversion prediction. Currently, various methods have been used to learn the common latent subspace across different modalities, such as canonical correlation analysis (CCA) (Chaudhuri et al., 2009). For AD diagnosis, the recent studies (Zhu et al., 2016a) have proposed to learn a common hidden subspace from the original feature space of the different modalities by canonical correlation analysis. Although significant progress has been achieved, existing multimodal methods for AD/MCI diagnosis seldom consider the negative influence of noise information conveyed in multimodal data, leading to sub-optimal performance.

Another common challenge in automated AD/MCI diagnosis is that those multimodal data are usually incomplete in a block-wise manner, where a specific modality (e.g., PET) may be absent for a subject. For instance, even though all subjects in the baseline Alzheimer's Disease Neuroimaging Initiative (ADNI) database have MRI data, only about half of subjects have PET and CSF data. The incomplete data problem may be caused by poor data quality, high cost of PET scanning, and patient dropouts. Since the collection of CSF requires invasive tests (e.g., lumbar puncture), this may deter the patient's commitment. To effectively use these incomplete multimodal data, existing studies have developed various methods to use these data for the diagnosis of AD and MCI, including (1) sample exclusion methods, (2) data imputation methods, and (3) multi-view learning methods.

In the first category, subjects with missing values are directly excluded (Friedman et al., 2001), which will significantly decrease the number of samples for model training and consequently degenerate the learning performance. In the second category, one employs a specific algorithm to impute missing values based on observed instances. There are various algorithms for data imputation, such as zero, k-nearest neighbor (Hastie et al., 1999), expectation maximization (Schneider, 2001), and singular value decomposition (Golub and Reinsch, 1970). Even though one can make use of all available samples after data imputation, these kinds of approaches usually introduce extra noise information in the data imputation process, thus degrading the robustness of the learned models.

In the third category, multi-view learning methods (Xiang et al., 2014; Liu et al., 2015a, 2017; Zhou et al., 2019) have been developed to directly use all available subjects (even though they may contain missing modalities), without discarding or imputing missing values. In multi-view learning methods, each view is usually treated as a specific data modality, and all training samples can be categorized into multiple groups based on the availability of the modality combinations. A multivariate classification method is proposed (Fan et al., 2008), which uses a regional statistical feature extraction scheme to extract the voxel shape and direction of the brain image. The features are captured in the functional structure representation, and then mixes feature selection methods and nonlinear support vector machines are used to classify brain abnormalities, but this method cannot effectively simulate the subtle and complex spatial structure of the brain. A high-dimensional spatial pattern classification model is proposed in Fan et al. (2007). This technology can identify subtle changes and complex spatial structure patterns within the brain, which can classify individuals with certain specificity and sensitivity. This method uses a feature selection mechanism to find the most relevant local clusters among multi-view data, and uses these local clusters to train a nonlinear support vector machine classifier using Gaussian kernels. But this method will cause a lot of loss of relevant information. A manifold-regularized multi-task feature learning method is proposed in Jie et al. (2015, 2016) to retain the inherent correlation between multiple data forms and the data distribution information in each form. Specifically, the feature learning on each modal is expressed as a single task, and the group sparsity regularizer is used to capture the internal correlation between multiple tasks (i.e., modalities), and common features are selected from multiple tasks. This method does not consider the influence of noise data on the experimental results. A method of multi-modal image registration by maximizing mutual information quantitative–qualitative measurement is proposed in Luan et al. (2008). By using the concept of quantitative and qualitative information measurement of events, quantitative-qualitative measure of mutual information (Q-MI) merges utility information into two images and it can make the registration process focus more on matching voxels with more efficient use value. Wu et al. (2008) proposed a learning-based deformable registration method. This method selects a set of geometric features with the best scale for each point in the brain by optimizing the energy function, which will transform the image with salient and consistent features. Points (between different individuals) are used for the initial registration of the two images, while other less significant and consistent points are added to the registration process. But this method will cause loss of image information. Laterly, Jia et al. (2010) and Wu et al. (2013) also proposed other methods for multi-modal image registration. Existing multi-view learning method only consider the pairwise relationship rather than high-order relationship among samples, while the exact relationship is more complicated than pairwise in practice.

**Figure 1:**
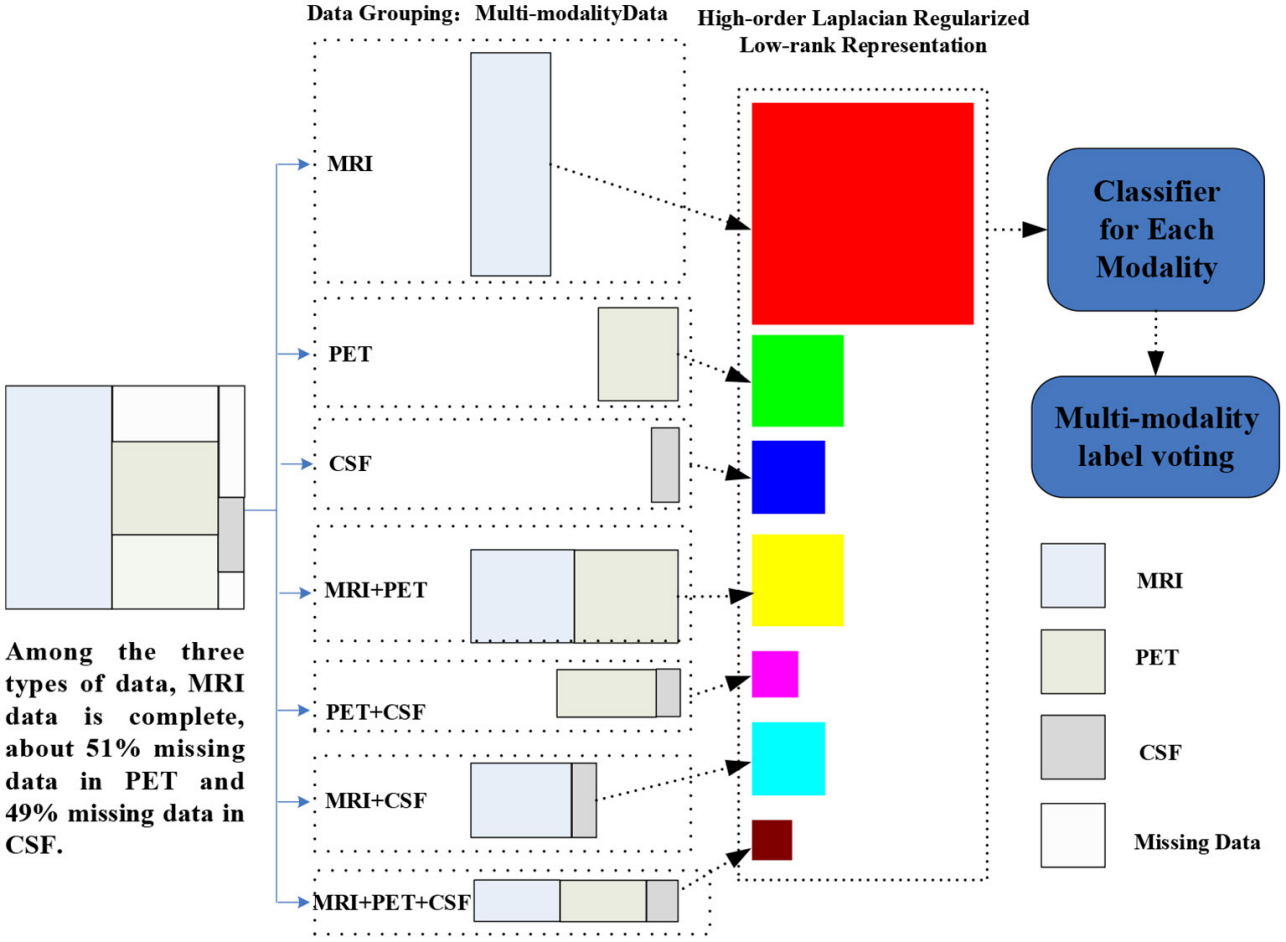
Illustration of the proposed hLRR method. Three main components included are as follows: (1) multimodal data grouping, (2) representation learning via the proposed hLRR model, and (3) ensemble classification based on the new representation.

To address these two issues, in this paper, we propose a high-order Laplacian regularized low-rank representation (hLRR) method for automated AD/MCI diagnosis based on incomplete heterogeneous multimodal data (i.e., MRI, PET, and CSF), as shown in Figure 1. Our method has the following contributions: (1) we propose an improved low-rank representation algorithm to remove the noise information of the original data; (2) we introduce a high-order Laplacian graph into the algorithm, and use the graph matrix to learn the structural relationships between and within multi-view data. The proposed method can help reduce the negative effect of the noise data via a low-rank constraint, and also can capture the high-order relationship among subjects via a hypergraph Laplacian regularization term.

The remainder of this paper is organized as follows. In section 2, we review the related work on brain disease diagnosis using incomplete multimodal data. In section 3, we present the materials used in this work and the proposed method in detail. Section 4 presents the experimental results on the public ADNI database. More discussions are included in section 5. We finally conclude this paper in section 6.

## 2. Related Work

### 2.1. Brain Disease Diagnosis With Multimodal Data

Based on multimodal data, the automated diagnosis of brain diseases (such as AD and MCI) is a very critical problem in the field because of the incomplete and heterogeneous data. Many studies consider multimodal data classification in a multi-view learning manner by treating each modality as one single view (Sun, 2013). Multi-view learning has attracted extensive attention due to its effectiveness in exploring complementary information among multiple views. In the early years, many researchers proposed a method based on the co-training (Blum and Mitchell, 1998) framework and the success and appropriateness of the method were also proved (Wang and Zhou, 2007). Recently, many researchers proposed an approach based on common hidden subspace learning using canonical correlation analysis (Kakade and Foster, 2007). For AD diagnosis, there is also some work (Xiang et al., 2014; Liu et al., 2017) aiming at taking full advantages of multimodal incomplete heterogeneous data.

Recent studies focus on addressing the multimodal problem through feature fusion by mapping multimodal data to a latent feature representation space. For instance, the deep constrained Boltzmann (Suk et al., 2014) was used to map multimodal data to a high-dimensional space for automated AD diagnosis. In Liu et al. (2018), high-level features in high dimensional space are generated to complete the classification task. In Zhu et al. (2016b), multimodal data are mapped into a unified feature space for feature selection. The sparse representation (Wang H. et al., 2011; Xu et al., 2015) is used to map multimodal data to a unified representation space. Although this method can ultimately retain the pathological information in the multimodal data, it can suppress the association between these multiple modalities. Several methods perform feature fusion by adding linear or nonlinear constraints to multimodal data. Depth polynomial network (DPN) (Shi et al., 2018) was used to add linear constraints on multimodal data for feature fusion to realize the diagnosis of AD. A new deep learning structure (Suk et al., 2016) was proposed to achieve feature fusion and AD detection by continuously weighting the feature information. However, existing methods seldom consider the problem of noise data and redundant information in multimodal data.

### 2.2. Low-Rank Representation

Low-rank representation (LRR) (Liu et al., 2013) is a well-known method employed to explore the potential low-dimensional subspace structure embedded in data. Currently, LRR techniques have attracted extensive attention in signal processing (Anvari et al., 2017), image processing (Du et al., 2017), computer vision (Zhou et al., 2011), and pattern recognition (Tan et al., 2010). Denote *N* as the number of samples, and *D* as the feature dimension. Given the *m*th modality data matrix ${\text{X}}_{\text{m}}=[{\text{x}}_{1},{\text{x}}_{2},\cdots ,{\text{x}}_{\text{i}},\cdots ,{\text{x}}_{N_{m}}]\in {\mathbb{R}}^{D_{M}\times N_{m}}$, the goal of LRR is to learn the lowest-rank representation to represent the data samples as linear combinations of the bases in a given dictionary. A classical LRR model is defined as follows:

(1) $$\arg\;\underset{\text{Z},\text{E}}{\min}\;rank(\text{Z})+\text{λ}\|\text{E}{\|}_{0}\;s.t.\;\text{X}=\text{A}Z+\text{E}$$

where **A** is a dictionary matrix and **E** denotes the error component. The term ‖**E**‖_0_ is the number of non-zeros in error matrix **E**, λ is a parameter to balance the lowest-rank representation and the error components. From Equation (1), we not only can obtain the low-rank representation of sample **X** but also can identify the noise information **E**. If we define the dictionary matrix **A** as sample matrix **X** itself, Equation (1) can be rewritten as follows:

(2) $$\arg\;\underset{\text{Z},\text{E}}{\min}\;rank(\text{Z})+\text{λ}\|\text{E}{\|}_{0}\;s.t.\;\text{X}=\text{X}Z+\text{E}$$

which can be rewritten as follows:

(3) $$\arg\;\underset{\text{Z},\text{E}}{\min}\|\text{Z}{\|}_{*}+\text{λ}\|\text{E}{\|}_{1}\;s.t.\;\text{X}=\text{X}Z+\text{E}$$

where ‖**Z**‖_*_ represents the nuclear norm the definition of which is the sum of all singular values of matrix **Z**, and ‖**Z**‖_*_ is the convex envelope of *rank*(**Z**) in Equation (2). The term ‖**E**‖_1_ is the *L*_1_ norm whose definition is the sum of absolutes of all entries and ‖**E**‖_1_ is the convex envelope of ‖**E**‖_0_ in Equation (2). In Equation (3), the matrix **Z** can be called an affinity matrix because of its element *z*_(_*i, j*) is a reflection of the similarity between *x*_*i*_ and *x*_*j*_. The column *z*_*i*_ of matrix **Z** can be used as a representation of sample *x*_*i*_ because *z*_*i*_ is a new representation of sample *x*_*i*_ in terms of other samples of **X**.

Many studies (Kim and Park, 2008; Wright et al., 2010) have imposed some other helpful regularizations on the matrix **Z** to introduce richer information. For example, if sparsity and nonnegative constraints are imposed over matrix **Z**, Equation (3) can be rewritten as follows:

(4) $$\begin{array}{l} \arg\;\underset{\text{Z},\text{E}}{\min}\|\text{Z}{\|}_{*}+\lambda \|\text{Z}{\|}_{1}+\gamma \|\text{E}{\|}_{1}\\ \;s.t.\;\text{X}=\text{X}Z+\text{E},\;\text{Z}>=0 \end{array}$$

from which one can observe that low-rank representation can capture the underlying low dimensional data structure. However, existing LRR methods do not explicitly consider the high-order relationship among samples and cannot straightforwardly be applied to deal with the incomplete multimodal data. To this end, we propose an hLRR method for dementia diagnosis using incomplete multimodal data.

## 3. Materials and Methods

### 3.1. Materials and Data Pre-processing

In this work, multimodal data from the baseline ADNI database (Jack et al., 2008) are used. According to the Mini-Mental State Examination (MMSE) and other criteria, subjects in ADNI can be categorized into three groups: normal controls (NCs), MCI subjects, and AD subjects. In the baseline ADNI database, there are a total of 805 subjects, including 226 NCs, 393 MCI subjects, and 186 AD subjects. All subjects have at least one of three data modalities, including T1-weighted structural MRI, fluorodeoxyglucose PET, and CSF. Detailed description of ADNI can be found online^1^.

For both MRI and PET modalities, we extractvolumetric gray matter tissue inside pre-defined regions-of-interest (ROIs) as feature representations. To be specific, for MRI, we first apply the anterior commissure (AC)–posterior commissure (PC) correction to each MRI scan by using the MIPAV software package. Then, each MRI is re-sampled to have the same resolution 256 × 256 × 256, followed by intensity inhomogeneity correction using N3 algorithm (Sled, 1998) and skull stripping (Wang Y. et al., 2011). Manual editing is performed to ensure that both skull and dura are removed. We then remove the cerebellum by warping a labeled template to each skull-stripped image, and then segment the brain into three tissues (i.e., gray matter, white matter, and CSF) using FAST (Zhang et al., 2001). The anatomical automatic labeling (AAL) atlas (Tzourio-Mazoyer et al., 2002) (with 90 pre-defined ROIs in the cerebrum) are aligned to the native space of each subject using a deformable registration algorithm. For each subject, we finally extract the volumes of gray matter tissue inside 90 ROIs as features. Note that these features are normalized by the total intracranial volume that is estimated by the summation of gray matter, white matter, and CSF volumes from all those 90 ROIs. For PET scans, we align each PET image onto its corresponding MRI scan by using rigid registration. Then, we compute the mean intensity of each ROI in each PET image as feature representation. Three CSF biomarkers is used in this work, including amyloid *β* (A*β*42), CSF total tau (t-tau), as well as the CSF tau hyperphosphorylated at threonine 181 (p-tau). In this way, for each subject with complete multimodal data, it can be represented by a 183-dimensional feature vector, i.e., 90-dimensional MRI features, 90-dimensional PET features, and 3-dimensional CSF features. For clarity, we summarize the details of studied subjects and their feature representations in Table 1.

**Table 1:** Number of subjects and number of features used in this study.

	**MRI**	**PET**	**CSF**
Feature dimension	90	90	3
AD subjects	186	93	102
MCI subjects	393	201	190
NC subjects	226	101	112
Total subjects	805	395	404

### 3.2. Proposed Method

As illustrated in Figure 1, our proposed method contains three steps as follows: (1) multimodal data grouping, (2) high-order low-rank representation model construction, and (3) ensemble-based classification, with details given below.

#### 3.2.1. Multimodal Data Grouping

Based on the characteristic of subjects in ADNI, we partition all subjects into seven groups, including (1) subjects with MRI, PET, and CSF modalities; (2) subject with MRI and PET modalities; (3) subject with MRI and CSF modalities; (4) subject with PET and CSF data; (5) subjects with MRI; (6) subject with PET; and (7) subject with CSF data. Using such a partition strategy, we can ensure that each group will have complete data. Denote ${\text{X}}_{\text{m}}={\left\{{\text{x}}_{m}^{i}\right\}}_{i=1}^{N_{m}}$ as the data matrix for all training subjects with the *m*th (*m*=1,⋯ ,7) representation, and *N*_*m*_ is the number of subjects in the *m*th group. Let {$X_{1}\in {\mathbb{R}}^{D_{1}\times N_{1}},{\text{X}}_{2}\in {\mathbb{R}}^{D_{2}\times N_{2}},\cdots \;,{\text{X}}_{m}\in {\mathbb{R}}^{D_{m}\times N_{m}},\cdots \;,{\text{X}}_{M}\in {\mathbb{R}}^{D_{M}\times N_{M}}$} denote subjects in those *M*=7 groups, where *N*_*m*_ and *D*_*m*_ is number of subjects and feature dimension, respectively, for the *m*th group.

#### 3.2.2. High-Order Low-Rank Representation

To capture the high-order relationship among multiple modalities, we formulate the multimodal incomplete heterogeneous data classification as a hypergraph construction problem. The hypergraph is the generalized version of the traditional graph. Each edge contains more than two vertices in a hypergraph, while each edge in a traditional graph only contains only two vertices in the traditional graph. Therefore, the hypergraph can convey some high-order relationship among vertices, whereas traditional edge only demonstrates the pairwise relationship between two vertices. In this study, we use hypergraph to discover the high-order information among vertices (with each vertex denoting a specific subject). We construct seven hypergraphs since there are seven groups in this study, aiming to model the high-order relationship among subjects within each group. Before constructing the hypergraph, we give some notations about hypergraph, summarized in Table 2.

**Table 2:** Notations used in a hypergraph.

**Notation**	***Meaning***
$G^{m}=(V,\varepsilon^{m},w^{m})$	The **m**th hypergraph corresponding to the **m**th modality
V	The vertex set that contains N vertices
ε^**m**^	The hyperedge set corresponding to the **m**th modality
$W_{e}^{m}$	The weights set for hyper-edges *ε*^**m**^, $w_{e_{j}}^{m}$ being its element
$w_{e_{j}}^{m}$	The weight for hyperedge **e**_**j**_ in the **m**th modality
$N_{m}^{e}$	The number of hyperedges in the **m**th modality
$W^{m}in\in {\mathbb{R}}^{D_{1}\times N_{1}}$	Diagonal matrix of hyperedge weights in the **m**-th modality
$H^{m}in\in {\mathbb{R}}^{D_{1}\times N_{1}}$	The vertex-hyperedge incidence matrix in the **m**th modality
$D_{e}^{m}$	The hyper-edge matrix with diagonal entries corresponding to the degree of each hyperedge
$D_{v}^{m}$	The vertex degree matrix with diagonal entries corresponding to the degree of each vertex

Based on the notations, we define the (*v*_*i*_, *e*_*j*_)-entry of matrix **H**^*m*^ indicating the vertex *v*_*i*_ is affiliated with hyper-edge *e*_*j*_ as

(5) $$h_{v_{i},e_{j}}^{m}=\{\begin{array}{cc} 1, & \;if\;v_{i}\in e_{j}\\ 0, & otherwise \end{array}$$

The degree of a vertex *v*_*i*_ is defined as

(6) $$d_{v_{i}}^{m}=\begin{array}{c} \sum_{e_{j}\in \xi^{m}}w_{e_{j}}^{m}h_{v_{i},e_{j}}^{m} \end{array}$$

The degree of a hyper-edge *e*_*j*_ is defined as

(7) $$\delta_{e_{j}}^{m}=\begin{array}{c} \sum_{v_{i}\in v}h_{v_{i},e_{j}}^{m} \end{array}$$

Also, the hyper-Laplacian matrix is typically employed to discover the high-order information among vertices/subjects based on the constructed hypergraph. Previous studies usually construct a hypergraph by using the Euclidean distance to measure the similarity between samples. However, the Euclidean distance cannot utilize global structure information. Several studies (Wright et al., 2009; Qiao et al., 2010) have proved that sparse representation is not only effective in reflecting the global structure information but also robust to data noise. Therefore, in this work, we employ sparse representation for hyperedge construction.

For the *m*th group, its training samples can be defined as the data matrix ${\text{X}}_{m}=[{\text{x}}_{1},{\text{x}}_{2},\cdots ,{\text{x}}_{i},\cdots ,{\text{x}}_{N_{m}}]\in {\mathbb{R}}^{D_{M}\times N_{M}}$. Based on the sparse representation theory (Qiao et al., 2010), each ***x***_*i*_ can be linearly represented using as few as other samples, e.g., ${\text{x}}_{i}=\sum_{j\neq i}s_{ij}{\text{x}}_{j}$ is a sparse representation coefficient. The general objective function of sparse representation is as follows:

(8) $$\underset{{\text{s}}_{\text{i}}}{\min}\left\|{\text{x}}_{i}-\text{X}{\text{s}}_{i}\right\|+\beta {\left\|{\text{s}}_{i}\right\|}_{1}\;s.t.1=1^{T}{\text{s}}_{i}$$

where $s_{i}={\left[s_{i1},\cdots ,s_{i,i-1},0,\cdots s_{iN}\right]}^{T}$ is a weight vector, *β* is a regularization parameter used to control the sparsity of **s**_*i*_, and 1 ∈ ℝ^*N*^ is a vector with all ones. In Equation (8), the component *s*_*ij*_ of the weight vector **s**_*i*_ is employed to measure the significance of ***x***_*j*_ to ***x***_*i*_. With the sparsity constraint, one can encourage that each ***x***_*i*_ is associated with as few other samples as possible. Let **s**_*i*_ be the optimal weight vector of Equation (8), the sparse representation weight matrix corresponding to data matrix **X**_*m*_ can be defined as

(9) $$\text{S}={\left[{\text{s}}_{1},\cdots ,{\text{s}}_{\text{i}},\cdots ,{\text{s}}_{\text{N}}\right]}^{T}$$

Based on Equation (9), we rewrite Equation (5) as

(10) $$h_{v_{i},e_{j}}^{m}=\{\begin{array}{cc} \left|s_{ij}\right|, & \;if\;\left|s_{ij}\right|>\theta \\ 0, & otherwise \end{array}$$

where *θ* is a small threshold and *s*_*ij*_ is the element of **S** in Equation (9).

With the construction of the hypergraph and the notations in Table 2 and based on our previous work about hyper-graph learning (Liu et al., 2017), we define each hyper-Laplacian (Zhou et al., 2007) corresponding to each group as follows

(11) $$\begin{array}{r} {\text{L}}_{m}=\text{I}-{\left({\text{D}}_{v}^{m}\right)}^{-\frac{1}{2}}{\text{H}}_{m}{\text{W}}_{m}{\left({\text{D}}_{e}^{m}\right)}^{-1}{\left({\text{H}}_{m}\right)}^{T}{\left({\text{D}}_{v}^{m}\right)}^{-\frac{1}{2}} \end{array}$$

where **I** is an identity matrix.

Motivated by the graph-based manifold learning (Belkin and Niyogi, 2002) and the high-order relationship based on hyper-graph construction, we can incorporate a hyper-Laplacian regularization term into Equation (4) so that data points within the same hyper-edge are similar to each other. We weight the summation of pair-wise distance among given data points within each hyper-edge by $\frac{{\text{W}}_{e}^{m}}{{\text{D}}_{e}^{m}}$. The hyper-Laplacian regularized form of Equation (4) can be written as:

(12) $$\begin{array}{l} \underset{{\;}_{\text{Z},\text{E}}}{\min}\;{\left\|\text{Z}\right\|}_{*}+\text{λ}{\left\|\text{Z}\right\|}_{1}+\beta \begin{array}{c} \sum_{(i,j)\subset e\in \xi }{\left\|z_{i}-z_{j}\right\|}^{2}\frac{\text{W}(\text{e})}{\text{D}(\text{e})}+\gamma {\left\|\text{E}\right\|}_{1} \end{array}\\ \;s.t.\;\text{X}=\text{X}Z+\text{E}\text{Z}>0 \end{array}$$

Finally, with some algebraic manipulations, we can obtain the hyper-Laplacian regularized LRR model by the matrix form:

(13) $$\begin{array}{l} \underset{\text{Z},\text{E}}{\min}\;{\left\|\text{Z}\right\|}_{*}+\lambda {\left\|\text{Z}\right\|}_{1}+\beta \mathrm{tr}\left(\text{Z}{\text{L}}^{h}{\text{Z}}^{T}\right)+\gamma {\left\|\text{E}\right\|}_{1}\\ \;s.t\;\text{X}\;=\;\text{XZ}\;+\;\text{E}\;\text{Z}\text{ > }0 \end{array}$$

where the first term ‖**Z**‖_*_ encourages to obtain the low-rank representation of the original data matrix **X**, the second term ‖**Z**‖_1_ guarantees that the sparsity criterion can better capture the local structure around each data vector, and the third term tr (**ZL**^*h*^**Z**^*T*^) guarantees to reflect the high-order relationship among all the data. Also, the fourth term is an error component that is used to remove the noise information.

For the *m*th block (w.r.t., **X**_*m*_), we construct a hypergraph to capture the high-order relationship among subjects, yielding a hyper-Laplacian matrix **L**_*m*_. To learn a new representation **Z**_*m*_, we define the objective function of hLRR as follows:

(14) $$\begin{array}{l} \underset{{\text{Z}}_{m},{\text{E}}_{m}}{\min}\sum_{m=1}^{M}{\left\|{\text{Z}}_{m}\right\|}_{*}+\text{λ}\sum_{m=1}^{M}\eta_{m}{\left\|{\text{Z}}_{m}\right\|}_{1}\\ \;+\beta \sum_{m=1}^{M}\eta_{m}\mathrm{tr}\;({\text{Z}}_{m}{\text{L}}_{m}{\text{Z}}_{m}^{T})+\gamma \sum_{m=1}^{M}\eta_{m}{\left\|{\text{E}}_{m}\right\|}_{1}\\ s.t.\;{\text{X}}_{m}={\text{X}}_{m}{\text{Z}}_{m}+{\text{E}}_{m},{\text{Z}}_{m}\geq 0\;(m=1,2,\cdots ,M) \end{array}$$

where $\mathrm{tr}\left({\text{Z}}_{m}{\text{L}}_{m}{\text{Z}}_{m}^{T}\right)$ is the hyper-Laplacian regularized item for the *m*th group. Also, *λ*, *β*, and *γ* are penalty parameters to balance the three regularization terms. Note that *η*_*m*_ is an indicator vector to denote whether each subject is involved in the *m*-th group, i.e., *η*_*m*_(*i*,*i*)=1 if $x_{m}^{i}$ exists; and 0, otherwise.

We use the linearized ADM with adaptive penalty (Lin et al., 2011) to solve Equation (14). First an auxiliary variable **J**_*m*_ is introduced to make the objective function of Equation (14) separable and then Equation (14) is formulated as:

(15) $$\begin{array}{l} \underset{{\;}_{{\text{Z}}_{m},{\text{E}}_{m},{\text{J}}_{m}}}{\min}\;\sum_{m=1}^{M}\;{\left\|{\text{Z}}_{m}\right\|}_{*}+\text{λ}\sum_{m=1}^{M}\eta_{m}{\left\|{\text{J}}_{m}\right\|}_{1}+\beta \sum_{m=1}^{M}\eta_{m}\mathrm{tr}\left({\text{Z}}_{m}{\text{L}}_{m}{\text{Z}}_{m}^{T}\right)\\ \;+\gamma \sum_{m=1}^{M}\eta_{m}{\left\|{\text{E}}_{m}\right\|}_{1}\\ s.t.\;{\text{X}}_{m}={\text{X}}_{m}{\text{Z}}_{m}+{\text{E}}_{m},{\text{Z}}_{m}={\text{J}}_{m},\\ \;{\text{J}}_{m}\geq 0,\;m=1,2,\cdots ,M \end{array}$$

The augmented Lagrangian function of Equation (15) is as follows:

(16) $$\begin{array}{l} L\left({\text{Z}}_{m},{\text{E}}_{m},{\text{J}}_{m},{\text{G}}_{m},{\text{Q}}_{m}\right)\\ =\sum_{m=1}^{M}{\left\|{\text{Z}}_{m}\right\|}_{*}+\lambda \sum_{m=1}^{M}\eta_{m}{\left\|{\text{J}}_{m}\right\|}_{1}+\beta \sum_{m=1}^{M}\eta_{m}\mathrm{tr}\left({\text{Z}}_{m}{\text{L}}_{m}{\text{Z}}_{m}^{T}\right)\\ +\gamma \sum_{m=1}^{M}\eta_{m}{\left\|{\text{E}}_{m}\right\|}_{1}\\ +\sum_{m=1}^{M}\left(\langle {\text{G}}_{m},{\text{X}}_{m}-{\text{X}}_{m}{\text{Z}}_{m}-{\text{E}}_{m}\rangle +\langle {\text{Q}}_{m},{\text{Z}}_{m}-{\text{J}}_{m}\rangle \right)\\ +\frac{\mu }{2}\left({\left\|{\text{X}}_{m}-{\text{X}}_{m}{\text{Z}}_{m}-{\text{E}}_{m}\right\|}_{F}^{2}+\;{\left\|{\text{Z}}_{m}-{\text{J}}_{m}\right\|}_{F}^{2}\right) \end{array}$$

where **G**_*m*_ and **Q**_*m*_ are Lagrange multipliers and *μ* >0 is a penalty parameter.

#### 3.2.3. Optimization

There are three variables in Equation (16) to be optimized. In this work, we employ an alternating iterative optimization method to solve the proposed problem. Thus, two main steps are included in the following iterative procedure: (1) fix parameter {**E**_*m*_, **J**_*m*_} in Equation (16) and then optimize **Z**_*m*_; (2) fix parameter **Z**_*m*_ in Equation (16) and then optimize {**E**_*m*_, **J**_*m*_}. These two steps are executed iteratively until some conditions are satisfied. For optimizing Equation (16), two theorems are provided in the section of Supplementary Material. The detailed description of solving the hLRR is shown in Table 3.

**Table 3:** The proposed method hLRR.

**Algorithm: High-order Laplacian regularized low-rank representation (hLRR)**
*Input **:X***_**m**_,*λ*, *β*, *γ* and the number of nearest neighbors
*Output **:Z***_**m**_, ***E***_**m**_
*Initialization* :Compute hyper-Laplacian matrix ***L***_**m**_; $E_{m}^{0}=J_{m}^{0}=G_{m}^{0}=Q_{m}^{0}$=0, *λ*=0.05, *β*=2.0, *γ*=5.0, *ρ*_0_=3, *μ*_0_=10^−6^, $\mu_{m}ax=10^{6}$; *ϵ*_1_=$\epsilon_{2}=10^{-6}$, k=0
**Repeat:**
*Step* 1: Update $Z_{m}^{k}$ using Equation (17)
*Step* 2: k=k+1
*Step* 3: Update $E_{m}^{k}$ using Equation (21)
*Step* 4: Update $J_{m}^{k}$ using Equation (22)
*Step* 5: Update Lagrange multipliers ***G***_***m***_ and ***Q***_***m***_, $G_{m}^{k+1}$= $G_{m}^{k}$+*μ*_**k**_(***X***_***m***_-$X_{m}Z_{m}^{k+1}$-$E_{m}^{k+1}$), $Q_{m}^{k+1}$= $Q_{m}^{k}$*+μ*_**k**_($Z_{m}^{k+1}$-$J_{m}^{k+1}$)
*Step* 6: Update *μ*: *μ*_*k*+1_=min(*μ*_**max**_,*ρ*_**k**_*μ*_**k**_), if max{$\eta_{1}\|Z_{m}^{k+1}-Z_{m}^{k}\|$, $\mu_{k}\|J_{m}^{k+1}-J_{m}^{k}\|$, $\mu_{k}\|E_{m}^{k+1}-E_{m}^{k}\|$} *≤* ϵ_2_, *ρ*_**k**_=*ρ*_0_; otherwise *ρ*_**k**_=1
*Until*: max{$\left\|Z_{m}^{k+1}-Z_{m}^{k}\right\|$,$\left\|J_{m}^{k+1}-J_{m}^{k}\right\|$,$\left\|E_{m}^{k+1}-E_{m}^{k}\right\|$} ≤ ϵ_2_ and $\frac{\left\|X_{m}-X_{m}Z_{m}^{k+1}-E_{m}^{k+1}\right\|}{\left\|X_{m}\right\|}$ ≤ ϵ_1_

#### 3.2.4. Ensemble-Based Classification

In the third step, with the new data representation, we train seven support vector machine (SVM) classifiers, with each SVM corresponding to a specific group. Then, we use a simple majority voting strategy to combine the outputs of multiple SVMs to make a final decision. Specifically, we assume that the output for each modality is y_*i*_ (*i* = 1, 2, ⋯ , *M*) where y_*i*_=1 denotes the positive categorical label (e.g., AD) and y_*i*_=–1 denotes the negative categorical label (e.g., NC). The final result for a new test subject ***x***_*j*_ can be expressed as $f({\text{x}}_{\text{j}})=sign(\frac{1}{M}\sum_{i=1}^{M}{\text{y}}_{i})$.

## 4. Experiments

### 4.1. Experimental Setup

For the benchmark datasets, they have been partitioned using 10-fold cross-validation strategy, i.e., each dataset is first partitioned into the training set and the testing set with a ratio of 9:1. And then, a 10-fold inner cross-validation is done on the training set for parameter selection, in which 9-folds are taken as training and the remaining fold as validation. In the experiments, we perform three classification tasks, including AD vs. NC, AD vs. MCI, and NC vs. MCI. Seven metrics are used for performance evaluation, including the classification accuracy (ACC), sensitivity (SEN), specificity (SPE), balanced accuracy (BAC), positive predicted value (PPV), negative predictive value (NPV), and the area under the receiver operating characteristic curve (AUC).

### 4.2. Competing Methods

We compare the proposed method hLRR with four data computation techniques, i.e., zero (filling the missing data with all zeros), K-nearest neighbor (KNN) (Hastie et al., 1999), expectation maximization (EM) (Schneider, 2001), singular value decomposition (SVD) (Golub and Reinsch, 1970). To illustrate the superiority over the other multimodal data classification methods, the proposed hLRR method is also compared with the state-of-the-art methods: two incomplete multi-source feature learning methods (iMSF) (Yuan et al., 2012) with logistic loss (denoted as iMSF-1) and square loss (denoted as iMSF-2) and convolutional nonnegative matrix factorization (CH-CNMF) (Vaz et al., 2016), deep multi-kernel learning (DMKL) (Strobl and Visweswaran, 2013), stack autoencoder (SAE) (Xu et al., 2017), logistic regression(LR) (Cui et al., 2016), and feature attribute fusion(Attf) (Bosnic et al., 2020). For the compared methods that were not originally developed to solve the incomplete data problem, we use the KNN algorithm to impute missing values, because of its stable performance. These compared methods are briefly summarized as follows:

1. Zero: This method is also called the complement zero method which replaces all the missing values with 0.
2. KNN: It finds the nearest *k*1 neighbors, and fills the missing values by the mean feature value of these neighbors. In this work, we set *k*1 = 5.
3. EM: The EM is a popular iterative refinement algorithm. Each step of EM algorithm consists of an expectation step and a maximization step. The basic idea is to estimate an initial value of missing data and calculate the value of model parameters. The step E and step M are performed iteratively to update the estimated missing values until convergence.
4. SVD: It completes the data by calculating the similarity between complete data and missing data. First, the data is processed and decomposed to obtain its feature vector. Second, the characteristic were used to find the value most similar to the missing value and complete it.
5. iMSF-1: After the multimodal data are divided and the complete data of each modal is obtained, sparse learning is used to map the multimodal data into a shared space. A classification integrator is constructed by combining the data completion algorithm and this method.
6. iMSF-2: The method is divided into two stages. In the first stage, the multimodal data is projected into a shared feature space, in which the model scores containing missing data are contained. In the second stage, the missing values in the score matrix are estimated and the data are completed.
7. DMKL: It combines multi-core learning with deep learning and draw on the idea of multi-core learning to introduce adjustable hyperparameters. Multiple kernels are sequentially combined into a multilayer deep network, where each kernel has an associated weight value. It improves the multi-core learning method by successfully optimizing each multi-layer with multiple cores.
8. CH-CNMF: The algorithm decomposes the data matrix into a basic tensor containing the time pattern and an activation matrix indicating the moment when the time pattern appears. It is important that the time pattern corresponds closely to the observed data and represents a wide dynamic range.
9. SAE: The stack de-noising automatic encoder is used to represent the modal data (early fusion). Then, on the basis of learning features, a number of SVMs are used to predict and classify the data with the learned features.
10. LR: This method aims to obtain a linear classifier with a decision function. The training and predicting framework is the same as LIBSVM. It predicts the probability that the sample belongs to a certain class, rather than hard labels.
11. Attf: This method performed data fusion from both attribute and sample views (Bosnic et al., 2020). For attributes fusion, it is performed by enriching attributes of the base dataset with those of the secondary dataset. For sample fusion, it is performed by enriching the examples set of the base dataset with those of the secondary dataset, with more details given in Bosnic et al. (2020).

The parameters *λ*, *β*, and *γ* in Equation (14) are determined from the parameter set {10^−7^, 10^−6^, ⋯ , 10^7^} with grid search strategy. The parameter *k*1 for KNN is determined from {3, 5, 7, 9, 11, 15, 20}, the rank parameter for SVD is determined from {80, 85, 90, 95}, and the parameter λ for iMSF is chosen from {10^−4^, 10^−6^, ⋯ , 10^3^}. The kernel parameter selection range of DMKL is [0, 1], and here we choose 0.1. The SVM classifier uses default parameters. All the methods are carried out in MATLAB (R2016b) on a computer with Intel(R) Core (TM) i7-4510U 2.50 GHz CPU and 16 GB RAM.

### 4.3. Results and Analysis

Table 4 shows the results of 11 different methods in AD vs. NC classification. From the results, it can be seen that all the algorithms have achieved good results, and the accuracy is about 90%. Compared with other centralized algorithms, our algorithm has achieved the best results. Compared with several data completion/imputation methods (i.e., Zero, KNN, EM, and SVD), our algorithm has improved the accuracy results by about 3%. Compared with iMSF-1 and iMFS-2, our method significantly improves the AUC values by about 7%. Compared with the other two deep learning methods, our algorithm improves the accuracy by about 3%, and improves the accuracy by 7% compared with the feature fusion method. Compared with the algorithm of logistic regression and factorization, it is improved by about 8%. In particularly, our hLRR method can yield better results in terms of SPE, PPV, and AUC, indicating that our method effectively reduces the misdiagnosis rate.

**Table 4:** Results (%) of seven different methods in AD vs. NC classification.

**Method**	**AD vs. NC**						
	**ACC**	**SEN**	**SPE**	**BAC**	**PPV**	**NPV**	**AUC**
Zero	91.72	85.82	96.73	**95.21**	91.81	86.65	84.50
KNN	90.61	89.97	95.78	93.21	92.58	**94.54**	85.44
EM	90.05	83.56	91.27	88.63	89.46	88.37	87.79
SVD	91.49	89.65	90.54	90.14	92.78	86.65	90.15
iMSF-1	86.19	86.42	86.29	86.35	83.41	89.09	86.34
iMSF-2	88.57	86.14	90.45	88.29	87.36	89.70	88.30
DMKL	91.78	83.67	86.62	91.64	94.36	80.56	89.56
CH-CNMF	85.41	84.62	86.21	85.41	92.59	73.33	87.62
SAE	91.37	**93.26**	90.86	91.95	90.89	90.00	90.32
LR	85.32	87.63	96.47	85.32	89.65	83.64	85.21
Attf	87.01	93.56	90.47	83.67	88.69	87.50	89.54
hLRR (ours)	**94.42**	81.58	**97.82**	94.49	**96.36**	84.05	**95.01**

*Accuracy (ACC), sensitivity (SEN), specificity (SPE), balanced accuracy (BAC), positive predictedvalue (PPV), negative predictive value (NPV), the area under the receiver operating characteristic curve (AUC). Best values are in bold*.

Table 5 reports the comparison results of different methods in AD vs. MCI classification. It can be seen that our method has made a great improvement in terms of accuracy, which is about 9% higher than other methods. The improvement in terms of other metrics is also obvious, suggesting the effectiveness of hLRR in identifying MCI subject from AD.

**Table 5:** Results (%) of seven different methods in AD vs. MCI classification.

**Method**	**AD vs. MCI**						
	**ACC**	**SEN**	**SPE**	**BAC**	**PPV**	**NPV**	**AUC**
Zero	77.40	33.80	92.94	63.89	70.81	71.57	36.77
KNN	76.38	34.17	96.13	59.66	75.35	57.80	40.30
EM	76.12	36.95	94.24	61.26	70.21	73.61	38.73
SVD	76.17	26.32	**95.73**	62.42	71.93	69.44	37.58
iMSF-1	74.48	38.49	91.70	65.10	69.83	76.07	65.10
iMSF-2	75.34	38.84	92.35	65.60	71.03	76.34	65.60
DMKL	77.49	46.75	86.87	65.87	79.62	60.34	59.56
CH-CNMF	74.14	20.00	93.02	56.51	76.92	50.36	69.62
SAE	76.53	95.32	37.26	64.37	77.85	70.69	70.36
LR	67.24	23.37	67.28	54.63	67.24	66.84	65.71
Attf	79.67	42.10	66.34	78.39	72.91	79.86	80.38
hLRR (ours)	**88.40**	**99.50**	88.08	**93.79**	**87.77**	**97.75**	**81.65**

*Accuracy (ACC), sensitivity (SEN), specificity (SPE), balanced accuracy (BAC), positive predictedvalue (PPV), negative predictive value (NPV), the area under the receiver operating characteristic curve (AUC). Best values are in bold*.

Besides, we report the MCI vs. NC classification results achieved by different methods in Table 6. As can be seen that the overall results of different methods in MCI vs. NC classification are inferior to those in both tasks of AD vs. NC and AD vs. MCI classifications. The underlying reason could be that, since MCI is a prodromal stage of AD, brain dysfunction of MCI subjects may be mild compared to AD subjects, and thus its challenging to identify MCI subjects from NCs. On the other hand, results in Table 6 suggest that, compared with the other methods, the proposed hLRR method achieves the improvement of at least 10% in terms of ACC and AUC values. These results further demonstrate the effectiveness of the proposed method in automated brain dementia diagnosis. From Tables 5, 6, we can see that the proposed hLRR is superior to the other methods. The possible reason is that our hLRR not only can avoid the influence of noise data but also can reflect the high-order relationship among multiple modalities.

**Table 6:** Results (%) of seven different methods in MCI vs. NC classification.

**Method**	**MCI vs. NC**						
	**ACC**	**SEN**	**SPE**	**BAC**	**PPV**	**NPV**	**AUC**
Zero	65.78	60.61	67.73	59.31	70.36	56.11	56.34
KNN	61.55	49.75	**78.98**	66.62	**78.73**	54.11	55.21
EM	65.91	66.96	64.85	66.01	76.33	59.41	56.01
SVD	68.39	57.22	72.32	64.97	72.67	59.81	53.24
iMSF-1	72.90	82.17	57.52	69.21	76.86	64.10	63.32
iMSF-2	75.68	95.40	33.43	64.42	75.60	77.36	64.42
DMKL	75.41	71.07	57.14	83.56	66.67	79.07	63.58
CH-CNMF	72.13	47.62	85.31	66.31	35.32	75.56	67.62
SAE	75.81	83.67	53.95	70.86	63.26	77.28	65.67
LR	67.74	34.95	83.62	69.27	67.24	72.14	56.65
Attf	72.58	62.50	78.95	70.72	76.92	72.14	65.32
hLRR (ours)	**85.32**	**95.63**	72.96	**84.96**	67.51	**92.26**	**83.54**

*Accuracy (ACC), sensitivity (SEN), specificity (SPE), balanced accuracy (BAC), positive predictedvalue (PPV), negative predictive value (NPV), the area under the receiver operating characteristic curve (AUC). Best values are in bold*.

**Figure 2:**
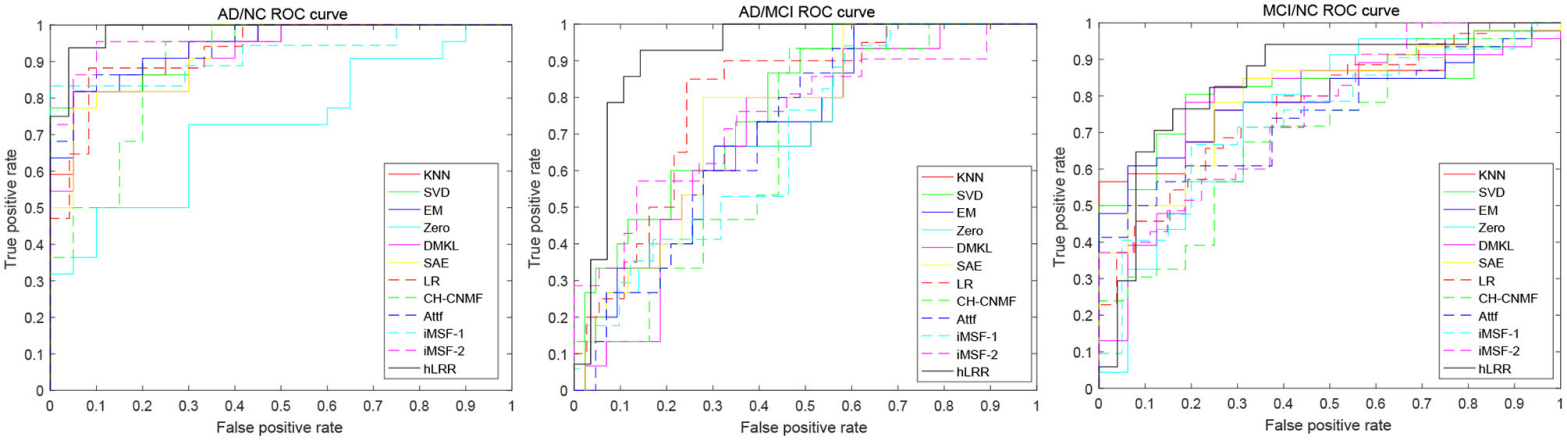
ROC curves achieved by different methods in **(left)** AD vs. NC classification, **(middle)** AD vs. MCI classification, and **(right)** MCI vs. NC classification.

Figure 2 shows the ROC curves of different methods in three tasks. Through the ROC curve diagram, we can clearly see that our method is significantly better than other methods in the detection of AD and its early symptoms, especially in the diagnosis of MCI. At the same time, our method reduces the results of false negative and false positive diagnosis errors. The diagnosis efficiency has been greatly improved, which provides a reliable guarantee for the early treatment of patients.

### 4.4. Computation Time

We now investigate the computational cost of the hLRR, and report the running time of different methods in AD vs. NC classification in Table 7. As can be observed from Table 7, the overall running time of our method is reasonable and acceptable in practical applications. But the proposed hLRR needs more running time compared to the other six methods, because of the time spent on the construction of multiple hypergraphs. In our future work, we will optimize the algorithm to reduce the time complexity.

**Table 7:** Running time of Zero, KNN, EM, SVD, iMSF-1, iMSF-2, DMKL, CH-CNMF, SAE, LR, Attf, and hLRR in AD vs. NC classification.

**Method**	**Zero**	**KNN**	**EM**	**SVD**	**iMSF-1**	**iMSF-2**	**DMKL**	**CH-CNMF**	**SAE**	**LR**	**Attf**	**hLRR (Ours)**
Time (s)	1.375	1.358	2.654	2.351	14.86	15.62	35.34	26.78	41.65	14.87	38.64	86.75

### 4.5. Parameter Analysis

**Figure 3:**
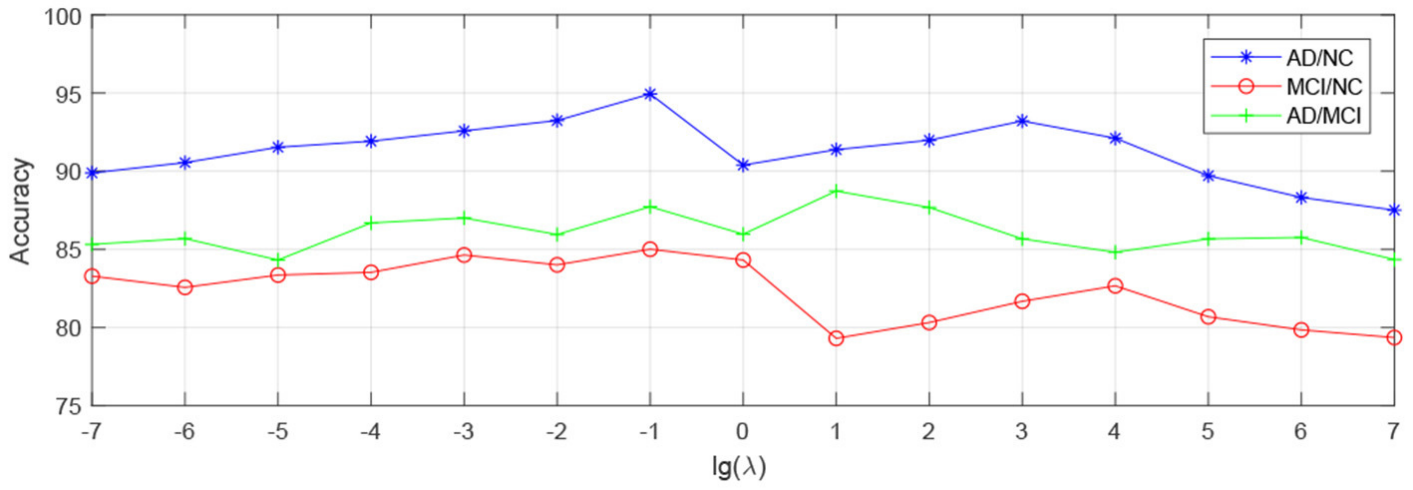
Influence of *λ* on hLRR (with *β* and *γ* fixed) in three classification tasks, i.e., AD vs. NC, MCI vs. NC, and AD vs. MCI classification.

**Figure 4:**
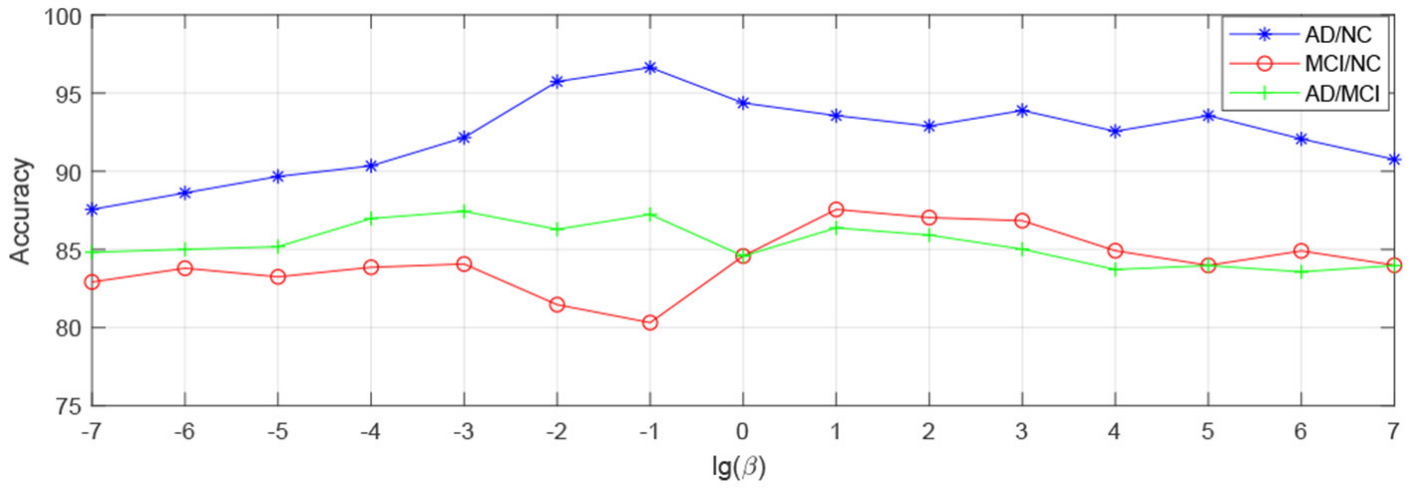
Influence of *β* on hLRR (with *λ* and *γ* fixed) in three classification tasks, i.e., AD vs. NC, MCI vs. NC, and AD vs. MCI classification.

**Figure 5:**
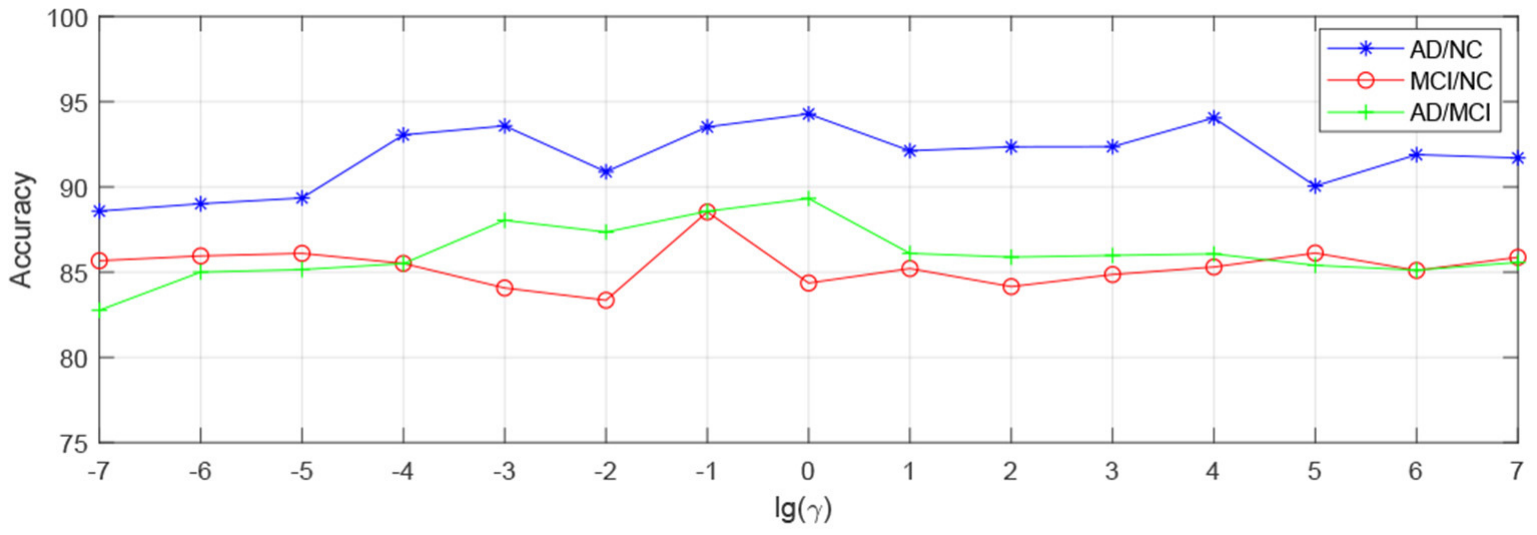
Influence of *γ* on hLRR (with *λ* and *β* fixed) in three classification tasks, i.e., AD vs. NC, MCI vs. NC, and AD vs. MCI classification.

In order to observe the sensitivity of a specified parameter (i.e., *λ*, *β*, and *γ*) in our method hLRR, we first fix all other parameters with their optimal values and then compare the performance of the proposed method under different values of this specified parameter. The experimental results on parameter sensitivity are reported in Figures 3–5. From Figure 3, we can see that, with the fixed *β*, and *γ*, the proposed hLRR can achieve the best result when λ = 0.1 for both tasks of AD vs. NC and MCI vs. NC classification, and the best result is achieved when λ = 10 for AD vs. MCI classification. The similar trend can be found in Figures 4, 5, that is, our hLRR method yields the best results when *β* and *γ* are within the range of [0.1, 10]. These results imply that the hLRR model is not very sensitive to three parameters. It can be seen from the experiments that the result is comparably good when the hyper-parameters are in domain [0.1, 10]. We will evaluate the proposed method on more datasets for extensive evaluation in the future.

### 4.6. Friedman and Nemenyi Test

**Figure 6:**
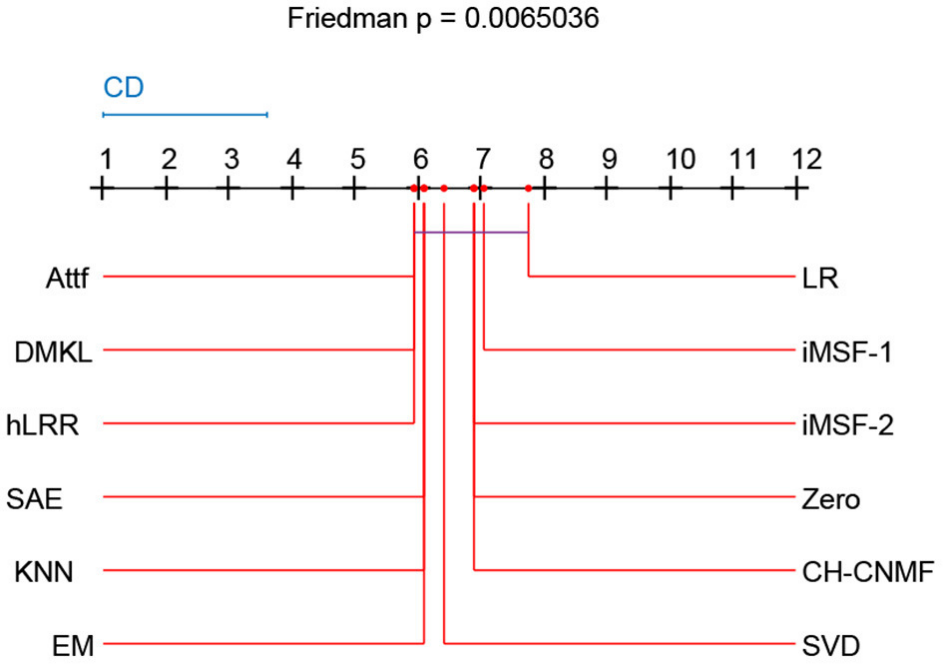
Nemenyi test chart of 12 different methods.

Friedman test and Nemenyi test are used to compare several algorithms on ADNI dataset. Friedman test can analyze whether there are obvious differences between all comparison algorithms on multiple datasets. Nemenyi test was used to further analyze whether those pairs of algorithms have significant differences. In Table 8, we report Friedman values for each algorithm in AD vs. NC classification. Figure 6 shows the nemenyi and algorithm difference diagram. From Table 8, we can clearly see that there are obvious differences between our algorithm and other algorithms. The gap between the theoretical value and the actual value of our algorithm is obviously better than that of other algorithms. According to Figure 6, we can intuitively see the difference between the two algorithms. The horizontal line in Figure 6 indicates the size of the average order value. The solid dot on the horizontal line represents the average order value of each corresponding algorithm. The blue line represents the size of CD value. The red line represents the CD value of each algorithm. The more overlapping red lines, the more similar the performance of the two algorithms. We can see that our hLRR has the same average order value as Attf and DMKL algorithm, and the red line has the highest overlapping degree, which indicates that three methods are roughly consistent in performance. Also, our hLRR has similar performance with SAE, KNN, and EM, and has a very big gap with other algorithms in performance. From Figure 6, we can clearly see that our method has achieved excellent results compared with other methods.

**Table 8:** Friedman values for 12 different methods.

**Method**	**Zero**	**KNN**	**EM**	**SVD**	**iMSF-1**	**iMSF-2**	**DMKL**	**CH-CNMF**	**SAE**	**LR**	**Attf**	**hLRR (Ours)**
Chi-sq	1686.72	1686.72	1409.97	553.55	1463.67	1576.96	1769.32	28700	1252.70	1569.79	1638.24	219.9

## 5. Discussion

### 5.1. Significance

Multimodality studies usually have to face the problem of missing modalities caused by patient dropouts or poor data quality. Existing AD-related studies typically discard modality-missing subjects, thereby greatly reducing sample size and degrading diagnostic performance (Zhang and Shen, 2012; Jie et al., 2016; Shi et al., 2020). This will significantly limit their utility in applications where subjects may usually lack one or several modalities. To this end, we propose an hLRR method for AD diagnosis and MCI conversion prediction based on incomplete multimodal data. Our method can fully make use of all subjects even those with missing modalities. It is worth noting that the proposed method can be straightforwardly applied to multimodality-based diagnosis of other brain diseases such as autism spectrum disorder (Wang et al., 2019; Lord et al., 2020) and Parkinson's disease (Bowman et al., 2016; Horsager et al., 2020).

### 5.2. Limitations and Future Work

Several limitations of the current work need to be considered. *On the one hand*, we simply employ the sparse representation technique to construct hypergraphs in this work, leading to a relatively higher computation cost. In the future, we will use other computationally light methods (e.g., k-nearest neighbor) to construct hypergraphs. *On the other hand*, we only evaluate our method on the ADNI database with MRI, PET, and CSF data. As future work, we will evaluate the proposed method on more new neuroscience applications or datasets, such as epileptic EEG recognition and Chinese physiological signal challenge dataset on electrocardiogram classification.

## 6. Conclusion

In this paper, we propose an hLRR method for brain dementia diagnosis using incomplete multimodal data. We first partition subjects into seven groups, with each group only containing modality-complete subjects. Then, we develop an hLRR learning model to capture the high-order relationship among subjects, with each hypergraph corresponding to a specific group. Based on the learned feature representations in multiple groups, we train multiple SVM classifiers, followed by an ensemble classification strategy to combine the outputs of different SVMs to make a final decision. Experimental results on the public ADNI database demonstrate the effectiveness of our proposed method for brain disease diagnosis.

## Data Availability Statement

Publicly available datasets were analyzed in this study. This data can be found at: http://adni.loni.usc.edu/.

## Author's Note

This work was finished when AD was visiting the University of North Carolina at Chapel Hill.

## Author Contributions

ML developed the theoretical framework and model in this work. AD implemented the algorithm. AD, ZL, and MW performed the experiments and result analysis. All authors drafted and revised the manuscript.

## Conflict of Interest

DS was employed by Shanghai United Imaging Intelligence Co., Ltd., Shanghai, China. The remaining authors declare that the research was conducted in the absence of any commercial or financial relationships that could be construed as a potential conflict of interest.

## Footnotes

^1^http://adni.loni.usc.edu.

## Appendix

**Theorem** 1. Suppose that {**E**_*m*_, **J**_*m*_} are fixed; then **Z**_*m*_ can be optimized with the following update rule:

(A1) $${\left({\text{Z}}_{m}^{k+1}\right)}^{*}=\Theta_{{\left(\eta_{1}\right)}^{-1}}\left({\text{Z}}_{m}^{k}-\nabla_{\text{z}}q\left({\text{Z}}_{m}^{k}\right)/\eta_{1}\right)$$

where Θ_ϵ_ (**A**)=**US**_ϵ_ (∑)**V**^*T*^ is the singular value thresholding operator (SVT) (Cai et al., 2010), $\text{U}{\text{S}}_{\epsilon }{\text{V}}^{T}$ is the singular value decomposition(SVD) of **A**, **S**_ϵ_ (*x*)=sgn(*x*) max(|*x*|-ϵ,0) is the soft thresholding operator.

**Proof**. With {**E**_*m*_, **J**_*m*_} fixed, Equation (16) can be expressed as

(A2) $$\begin{array}{l} L\left({\text{Z}}_{m},{\text{G}}_{m}^{k},{\text{Q}}_{m}^{k}\right)=\sum_{m=1}^{M}{\left\|{\text{Z}}_{m}\right\|}_{*}+\beta \sum_{m=1}^{M}\eta_{m}\mathrm{tr}\left({\text{Z}}_{m}{\text{L}}_{m}{\text{Z}}_{m}^{T}\right)\\ +\frac{\mu }{2}\sum_{m=1}^{M}\left({\left\|{\text{Z}}_{m}-{\text{J}}_{m}^{k}+\frac{1}{\mu }{\text{Q}}_{m}^{k}\right\|}_{F}^{2}\right)\\ +\frac{\mu }{2}\sum_{m=1}^{M}\left({\left\|{\text{X}}_{m}-{\text{X}}_{m}{\text{Z}}_{m}-{\text{E}}_{m}^{k}+\frac{1}{\mu }{\text{G}}_{m}^{k}\right\|}_{F}^{2}\right) \end{array}$$

Since Equation (A7) does not have a closed-form solution, following Lin et al. (2011), we denote the smooth component of Equation (A7) with

(A3) $$\begin{array}{l} q\left({\text{Z}}_{m},{\text{E}}_{m}^{k},{\text{J}}_{m}^{k},{\text{G}}_{m}^{k},{\text{Q}}_{m}^{k}\right)=\beta \;\sum_{m=1}^{M}\eta_{m}\mathrm{tr}\;\left({\text{Z}}_{m}{\text{L}}_{m}{\text{Z}}_{m}^{T}\right)\\ +\frac{\mu }{2}\sum_{m=1}^{M}\left({\left\|{\text{Z}}_{m}-{\text{J}}_{m}^{k}+\frac{1}{\mu }{\text{Q}}_{m}^{k}\right\|}_{F}^{2}\right)\\ +\frac{\mu }{2}\sum_{m=1}^{M}\left({\left\|{\text{X}}_{m}-{\text{X}}_{m}{\text{Z}}_{m}-{\text{E}}_{m}^{k}+\frac{1}{\mu }{\text{G}}_{m}^{k}\right\|}_{F}^{2}\right) \end{array}$$

According to linearized ADM with adaptive penalty (Lin et al., 2011), Equation (A3) can be approximated by its linearization $\sum_{m=1}^{M}\langle \nabla_{z}q({\text{Z}}_{m}^{k}),{\text{Z}}_{m}-{\text{Z}}_{m}^{k}\rangle$ plus a proximal term $\frac{\eta_{1}}{2}\sum_{m=1}^{M}{\left\|Z_{m}-Z_{m}^{k}\right\|}_{F}^{2}$, as long as $\eta_{1}\;>\;2\beta \;{\left\|L\right\|}_{2}+\mu (1\;+\;{\left\|X\right\|}_{F}^{2})$, among which $\nabla_{z}q\left({\text{Z}}_{m}^{k}\right)$ is the gradient of q w.r.t., ${\text{Z}}_{m}^{k}$. Equation (7) is equivalent to the following equation:

(A4) $$\begin{array}{l} \underset{{\text{Z}}_{m}}{\text{min}}\sum_{m=1}^{M}\|{\text{Z}}_{m}{\|}_{*}+\sum_{m=1}^{M}\langle \nabla_{\text{z}}q\left({\text{Z}}_{m}^{k}\right),{\text{Z}}_{m}\\ -{\text{Z}}_{m}^{k}\rangle +\frac{\eta_{1}}{2}\sum_{m=1}^{M}{\left\|{\text{Z}}_{m}-{\text{Z}}_{m}^{k}\right\|}_{F}^{2} \end{array}$$

with a closed-form solution given by Equation (A1).

**Theorem** 2. Suppose that **Z**_*m*_ is fixed; then {**E**_*m*_, **J**_*m*_} can be optimized with the following update rules, respectively.

(A5) $${\left({\text{E}}_{m}^{k+1}\right)}^{*}=S_{\frac{\gamma }{\mu }}\left({\text{X}}_{m}-{\text{X}}_{m}{\text{Z}}_{m}^{k+1}+\frac{1}{\mu }{\text{G}}_{m}^{k}\right)$$

(A6) $${\left({\text{J}}_{m}^{k+1}\right)}^{*}=\max\left\{{\text{S}}_{\frac{\gamma }{\mu }}\left({\text{Z}}_{m}^{k+1}+\frac{1}{\mu }{\text{Q}}_{m}^{k},0\right)\right\}$$

**Proof**. Suppose that **Z**_*m*_ is fixed, then we view {**E**_*m*_, **J**_*m*_} as a larger block of variables. We can update {**E**_*m*_, **J**_*m*_} independently by minimizing Equation (16), which naturally splits into two sub-problems for **E**_*m*_ and **J**_*m*_, respectively. So the problem for minimizing **E**_*m*_ is formulated as follows:

(A7) $$\begin{array}{l} L_{2}\left({\text{E}}_{m},{\text{G}}_{m}^{k}\right)=\gamma \sum_{m=1}^{M}\eta_{m}{\left\|{\text{E}}_{m}\right\|}_{1}+\frac{\mu }{2}\sum_{m=1}^{M}(\|{\text{E}}_{m}\\ {-\left({\text{X}}_{m}-{\text{X}}_{m}{\text{Z}}_{m}+\frac{1}{\mu }{\text{G}}_{m}^{k}\right)\|}_{F}^{2} \end{array}$$

The problem for minimizing **J**_*m*_ is formulated as follows:

(A8) $$L_{3}\left({\text{J}}_{m},{\text{M}}_{m}^{k}\right)=\lambda \sum_{m=1}^{M}{\left\|{\text{J}}_{m}\right\|}_{1}+\frac{\mu }{2}\sum_{m=1}^{M}\left({\left\|{\text{J}}_{m}-\left({\text{Z}}_{m}+\frac{1}{\mu }{\text{Q}}_{m}^{k}\right)\right\|}_{F}^{2}\right)$$

Obviously, Equations (A7) and (A8) has the closed-form solution as Equations (A5) and (A6), respectively.
